# Supplementary material for: Reconciling Mining with the Conservation of Cave Biodiversity: A Quantitative Baseline to Help Establish Conservation Priorities
Source: PLoS One. 2016 Dec 20;11(12):e0168348. doi: 10.1371/journal.pone.0168348 (PMC5173368; doi:10.1371/journal.pone.0168348)
Supplement: S1 Dataset — (ZIP) [file pone.0168348.s002.zip › Taxa/Serra Norte/SN_2013/N4E/Lista N4E-94.pdf]

CAVIDADE N4E-0094

Classe	Ordem	Fam/Outros	Gên/Outros	Espécie	S-U	U-S	Zon.
Arachnida	Acari	Gamasida		jovem		X	E
Arachnida	Amblypygi	Phrynidae	*Heterophrynus*	*longicornis*	1	X	E
Arachnida	Araneae	Ochyroceratidae		jovem		X	E
Arachnida	Araneae	Pholcidae	*Mesabolivar*	aff. *cambridgei*	X		E
Arachnida	Araneae	Pholcidae	*Mesabolivar*	*aurantiacus*		X	E
Arachnida	Araneae	Pholcidae	*Mesabolivar*	sp.1	X	X	E
Arachnida	Opiliones	Stygnidae	*Protimesius*	*gracilis*	1		E
Chilopoda	Scolopendromorpha	Scolopocryptopidae	*Newportia*	jovem		1	E
Entognatha	Collembola	Paronellidae		sp.1	X		E
Entognatha	Collembola	Sminthuroidea		sp.2		X	E
Entognatha	Diplura	Campodeidae		sp.1	X	X	E
Insecta	Coleoptera	Carabidae		sp.3	X	X	E
Insecta	Coleoptera	Staphylinidae	Pselaphinae	sp.35	X		E
Insecta	Coleoptera	Staphylinidae	Pselaphinae	sp.4		X	E
Insecta	Coleoptera	Staphylinidae		sp.1		X	E
Insecta	Diptera	Chironomidae		sp.	X		E
Insecta	Diptera	Dolichopodidae		sp.	X		E
Insecta	Diptera	Tipulidae	Tipulinae	sp.	X		E
Insecta	Heteroptera	Cydnidae		jovem		X	E
Insecta	Hymenoptera	Formicidae	*Camponotus*	sp.1	X		E
Insecta	Hymenoptera	Formicidae	*Pheidole*	sp.3	X		E
Insecta	Lepidoptera	Noctuoidea		sp.5		2	E
Insecta	Lepidoptera			jovem	X	X	E
Insecta	Mantodea	Mantidae		sp.1	1		E
Insecta	Orthoptera	Phalangopsidae	*Paraclodes*	sp.	3		E
Insecta	Psocoptera			jovem		X	E
Gastropoda	Pulmonata	Systrophiidae	*Happia*	sp.	X		E
Symphyla		Scutigerellidae		sp.	X	X	E
Mammalia	Chiroptera	Emballonuridae	*Peropteryx*	*kappleri*	1		E
